# Sensitivity of a Ratio Vegetation Index Derived from Hyperspectral Remote Sensing to the Brown Planthopper Stress on Rice Plants

**DOI:** 10.3390/s19020375

**Published:** 2019-01-17

**Authors:** Ye Tan, Jia-Yi Sun, Bing Zhang, Meng Chen, Yu Liu, Xiang-Dong Liu

**Affiliations:** Department of Entomology, Nanjing Agricultural University, Nanjing 210095, China; 2015102081@njau.edu.cn (Y.T.); sjynjau@163.com (J.-Y.S.); 2016102078@njau.edu.cn (B.Z.); 2017102078@njau.edu.cn (M.C.); 2018102085@njau.edu.cn (Y.L.)

**Keywords:** *Nilaparvata lugens*, population size, reflectance, rice, sensitivity, spectral index, time threshold

## Abstract

Remote sensing end-products related to vegetation have potential applications in monitoring the health of crops. The sensitivity of a spectral index to crop stress determines its application prospect. Our aim in this study was to explore the sensitivity of a ratio vegetation index (RVI) to identify the damage caused by brown planthoppers (BPHs) on rice plants, and to evaluate the potential application of hyperspectral end-products to monitor population size of BPH. Different numbers of the second-instar nymphs were released onto potted rice at the tillering stage. The plants were exposed to BPH for two, four, six, and eight days, and reflectance from the damaged rice was measured using a hyperspectral spectroradiometer. Measurements were done again two, four, and six days after exposure (recover days), and then the spectral index RVI_746_/_670_ was compared among rice plants infested with different numbers of BPH. The relationships between RVI_746/670_, the number of BPH and exposure day were simulated by linear and curve models. BPH damage resulted in a decreased spectral index RVI_746_/_670_ of rice plants. RVI_746/670_ well indicated the damage of rice plants caused by six–eight BPH nymphs per plant in six–eight days, but the index could not identify the damage of these nymphs in two days. The RVI_746/670_ showed a two–four-day delay to indicate a slight BPH damage. The spectral index RVI_746_/_670_ could indicate the physiologic compensation of plants for the feeding of BPH and the post-effect of BPH damage on rice. The RVI_746_/_670_ of rice showed a quadratic curve relation with the number of BPH nymphs and a quadratic or linear relation with the exposure day. The recover day had no significant effects on RVI_746/670_. The RVI_746_/_670_ (Y) could be simulated by a quadratic surface model based on the number of BPH (N) and exposure day (T): Y = 3.09427 + 0.59111T + 0.44296N − 0.03683T^2^ − 0.03035N^2^ − 0.08188NT (R^2^ = 0.5228, *p* < 0.01). In summary, the spectral index RVI_746_/_670_ of rice is sensitive to damage caused by BPH.

## 1. Introduction

A slight change in physical structure or chemical substance of plant leaves would result in changes of their spectral characteristics, and therefore, the hyperspectral remote sensing end-products have the potential to reflect stresses imposed on crops [1,2,3,4]. Hyperspectral remote sensing techniques are fast, nondestructive and precise in monitoring changes of crops. Herbivore insects feed on crops that alter the leaf area, color, tissue structure, or content of chemical substance, so the feeding damages may be identified by hyperspectral traits of crops [5,6,7,8]. Hyperspectral sensing has a good prospect in monitoring insect pests on crops [9,10,11,12].

Many studies have found that there are constant relationships between spectral index from crops and pest damage levels [13,14,15]. The spectral reflectance in 400–900 nm from winter wheat damaged by Russian wheat aphids *Diuraphis noxia* was significantly lower than the healthy wheat without aphids [16]. The spectral reflectance of tobacco, especially in the infrared waveband ranges, decreased when tobacco leaves were suffered from aphid feeding [17]. Ratio vegetation index (RVI) and normalized differenced vegetation index (NDVI) that derived from spectral reflectance from crops were strongly related to the population size of insect pests on crops [6,12]. It has been found that the values of NDVI and Green NDVI from wheat were significant negatively related with the stress from the greenbug *Schizaphis graminum* [6], and this relationship had also been found on rice plants damaged by the brown planthopper (BPH) *Nilaparvata lugens* [12,18]. Moreover, the RVI_800/450_ and RVI_950/450_ were able to identify the damage of wheat from greenbugs and Russian wheat aphids [19]. The RVI_730/670_ from rice leaf and canopy was related to the infestation scale of the rice leaf folder *Cnaphalocrocis medinalis* [7], and the NDVI_755/664_ from rice canopy could identify the BPH damages [8]. In this study, we found that the RVI_746/670_ from rice leaves had the potential to distinguish the number of BPH on rice. The vegetation index RVI exhibited a great ability to indirectly indicate the contents of chlorophyll and nitrogen in leaves, leaf area, and dry weight [20], and it therefore has a great potential to assess the insect pest damage for crops.

While many studies under control and natural conditions have confirmed that the pest stress can result in the change of hyperspectral characteristics of crops [6,7,12], there are limited actual field applications of hyperspectral remote sensing to monitor pest damages. To our knowledge, the sensitivity of a hyperspectral index to pest stress on crops must be understood before the application of this index, but few studies have been made on this issue. The previous studies mainly focused on the relationship of a hyperspectral index with the damage level of crops at a specific time [3,4,5,6,7,8,9,10,11,12,13,14,15], but did not successively assess this relationship as the damage levels changed. Thus, the sensitivity of a hyperspectral index to crop damages was still unknown. BPH is a serious pest on rice plants that often causes great yield losses of rice [11,12]. Effective pest control needs the support of population monitoring and forecasting data to determine when the control will be carried out. Nowadays, the monitoring method in rice fields for BPHs is still dependent on manual labor to sample and survey, which cannot satisfy the requirements of the modern agriculture because of its high labor costs and relative low accuracy [21]. Therefore, in this study, we focused on the sensitivity of a hyperspectral index to the BPH damage on rice plants, in order to promote the hyperspectral remote sensing method to be used in monitoring pests in the field.

## 2. Materials and Methods

### 2.1. Rice Plants and Insects

The variety of rice was Wuyujing 7 which was round-grained rice. The rice plants at tillering stage were transplanted into plastic cups (diameter 84 mm, height 100 mm), and then kept at a constant temperature room (27 °C, light:dark = 12 h:12 h). BPHs were collected from rice fields in Nanjing, China and reared in a growth chamber at 27 °C, light:dark = 12 h:12 h using rice seedlings.

### 2.2. Transfer BPHs onto Rice Plants

**Figure 1:**
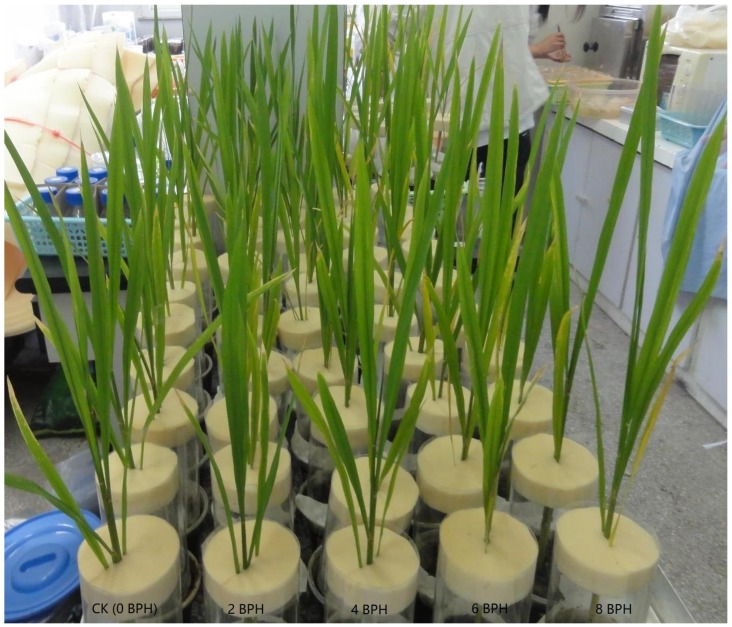
The rice plants damaged by 0~8 BPH 2rd-instar nymphs for 6 days.

In order to attain a series of homogeneous rice plants, the leaf area and SPAD readings of two expanded leaves in a plant were measured, and these rice plants with the similar leaf area (4.6~5.2 cm^2^) and SPAD readings (23.5~29.8) were chosen for the experiment. The leaf area was measured through multiplying the length by the max width of a leaf. The SPAD readings were measured by a chlorophyll meter (SPAD-502, Soil–Plant Analysis Development Section, Minolta Camera Co., Osaka, Japan). There were five leaves in a rice plant, and the three old leaves below were not measured, and only the two expanded leaves up were done (Figure 1).

The second-instar BPH nymphs were released onto 60 potted rice plants according to different population densities, and then the rice was covered by a transparent plastic chamber with a sponge plug to avoid the escape of nymphs (Figure 1). Five BPH densities 0, 2, 4, 6, and 8 nymphs per rice plant were designed. The rice plants were exposed to BPH for 2, 4, 6, and 8 days, and then all BPHs were removed. After 6 days exposure, there were no visible symptoms in rice plants exposed to 0, 2, 4, and 6 BPH nymphs, but 8 BPHs resulted in depigmentation of a part leaves (Figure 1). Spectral reflectance of rice plants in cups was measured at 15 cm above the leaves in 0, 2, 4, and 6 days after exposure (named recover day). The rice plants not exposed to BPH were control, named healthy rice. The experiments carried out three replications under a constant condition in the laboratory.

### 2.3. Measurement of Spectral Reflectance

The spectral reflectance of a rice plant was examined in a dark room at 25 °C using an ASD FieldSpec HandHeld Spectroradiometer (Analytical Spectral Devices, Inc., Boulder, CO, USA) and a 50 W halogen lamp. The spectral range is 325–1 075 nm with a resolution of 3 nm, and sampling interval is 1.6 nm. The field of view angle of the ASD radiometer is 25°. All leaves of a rice plant were placed on a piece of black cloth in order to reduce the effect of background on reflectance. The ASD was fixed 15 cm above the center of rice leaves, ensuring all leaves of a plant located in the field of the radiometer. The light source was fixed 40 cm away from leaves with a 45° angle. The ASD was preheated 20 min and then connected to the FieldSpec RS^2^ system to collect reflectance from rice leaves. Before recording, the radiometer was calibrated by a 91 mm round white reference panel that is nearly 100% reflecting and white referencing again every 15 min. A dark current measurement was applied when taking a white reference for reflectance measurements. The reflectance from a rice plant was collected successively ten times in the same field, and their average was considered as the reflectance of this rice plant.

### 2.4. Data Analysis

**Figure 2:**
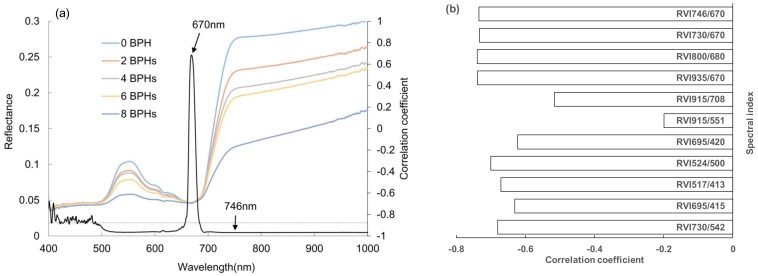
Reflectance from a rice plant exposed to 0–8 BPH nymphs for six days and the correlation coefficients (the black curve) between reflectance in each 1 nm wavelength from 400 to 1000 nm and the number of BPH (**a**), and the correlation between the ratio vegetation index and the number of BPH (**b**). The horizontal dash line in (**a**) represents the significant level of the correlation coefficient at *p* = 0.05.

The spectral data collected from rice plants were analyzed using the ASD software (View Spec Pro Version 5.0), and the reflectance in 325 to 1075 nm was attained from the rice plants. The reflectance reached to a high and stable level after 746 nm (Figure 2a). The correlations between the reflectance from a rice plant in each 1 nm waveband and the number of BPH feeding on this plant were analyzed, and the maximally positive and minimally negative correlation coefficients appeared at 670 nm and 746 nm, respectively (Figure 2a). Moreover, the ratio vegetation indices around 746 nm and 670 nm had the highest negative correlation with the number of BPH damaged rice plants (Figure 2b). Therefore, in this study, we chose these two wavebands to set up a ratio vegetation index RVI_746/670_ and assessed the sensitivity of this index to BPH damage. The RVI_746/670_ was computed by the ratio of reflectance at 746 nm (R_746_) to 670 nm (R_670_), RVI_746/670_ = R_746_/R_670_. The differences in RVI_746/670_ from rice plants damaged by different densities of BPH were analyzed by ANOVA followed the Post Hoc Tukey’s test. In order to explore the effects of BPH density, exposure day and recover day on RVI_746/670_, a repeated measure ANOVA was performed as the recover day was a repeated measure factor. The relationships between RVI_746/670_ and the BPH density or exposure day were simulated by a quadratic curve or linear function. The relationships between RVI, BPH density, and exposure day on a rice plant were simulated by a quadric surface model. The linear regression relation between estimated and measured RVI_746/670_ from rice was analyzed to evaluate the goodness of fit of the quadratic surface model. We used the *F* and *p* values to show the significant level. The *p*-value less than 0.05 was considered to be significant in all the statistics analyses. The simulation and statistics of data were performed using OriginPro 8 and SAS9.0 software, respectively.

## 3. Results

### 3.1. RVI746/670 of Rice Plants Exposed to BPH for Different Days

**Figure 3:**
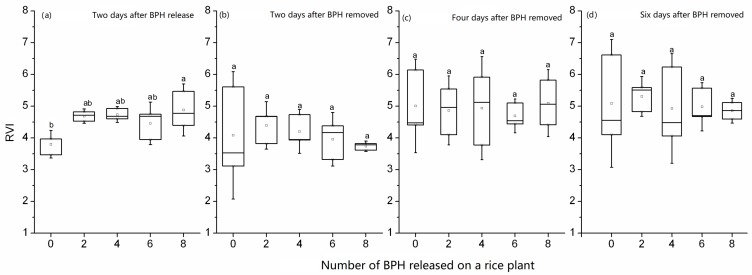
RVI_746/670_ of rice leaves exposed to 0–8 second-instar nymphs of BPH in two days (**a**) and 2–6 days after BPH exposure (**b**,**c**,**d**). In the box plot, the box represents the 25th and 75th percentiles; whiskers represent the minimum and maximum values; the line in box represents the median percentile; the open circle in a box represents the mean. The different letters above the box plots show significant differences in the RVI_746/670_ means among different numbers of BPH at *p* = 0.05 level.

The spectral index RVI_746/670_ of a rice plant at tillering stage increased when it was damaged by two–eight 2rd-instar nymphs for two days, and the value was significantly higher from the rice plant exposed to 8 BPH nymphs than from the rice plant without exposure (*F*_4,10_ = 4.27, *p* = 0.028), but there were no significant differences in values from rice plants exposed to two to eight nymphs (Figure 3a). Two–six days after BPH exposure, the RVI_746/670_ of rice plants damaged by two–eight nymphs in two days was not significantly different from the control without BPHs (2 days after exposure: *F*_4,10_ = 0.354, *p* = 0.836, Figure 3b; four days after exposure: *F*_4,10_ = 0.107, *p* = 0.977, Figure 3c; six days after exposure: *F*_4,10_ = 0.128, *p* = 0.969, Figure 3d). The overlapping ranges of the spectral index RVI_746/670_ values among different number of BPHs indicate that this spectral index is not sensitive to the damage of two–eight nymphs in two days (Figure 3).

**Figure 4:**
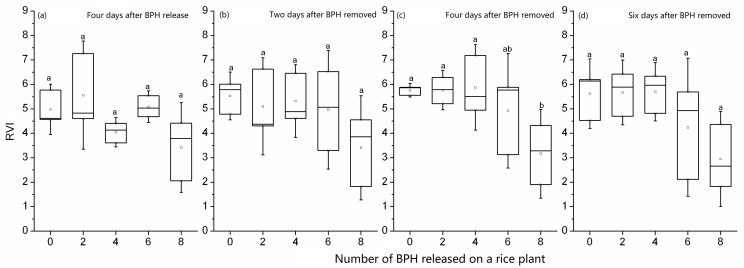
RVI_746/670_ of rice leaves exposed to 0–8 second-instar BPH nymphs for four days (**a**) and 2–6 days after BPH exposure (**b**,**c**,**d**). In the box plot, the box represents the 25th and 75th percentiles; whiskers represent the minimum and maximum values; the line in box represents the median percentile; the open circle in a box represents the mean. The different letters above the box plots show significant differences in the RVI_746/670_ means among different numbers of BPH at *p* = 0.05 level.

When the rice plants were exposed to zero–eight nymphs for four days, their RVI_746/670_ values were overlapping among different nymph densities (*F*_4, 10_ = 2.507, *p* = 0.109, Figure 4a). Moreover, two days after exposure, the RVI_746/670_ still had no differences among zero–eight nymphs (*F*_4, 10_ = 1.361, *p* = 0.314, Figure 4b). However, four days after exposure, the RVI_746/670_ from the rice plant exposed to 8 nymphs was significantly lower than that of the rice exposed to 0–4 nymphs, suggesting that the post-effect of BPH damage on rice plants was detected by the spectral index (*F*_4, 10_ = 3.527, *p* = 0.048, Figure 4c). Six days after exposure, the effect of BPH nymph’s damage on the spectral index RVI_746/670_ of rice plants was missed (*F*_4, 10_ = 2.970, *p*=0.074, Figure 4d). The spectral index RVI_746/670_ showed a four–day delay for indicating a short-term damage of rice plants by BPH.

**Figure 5:**
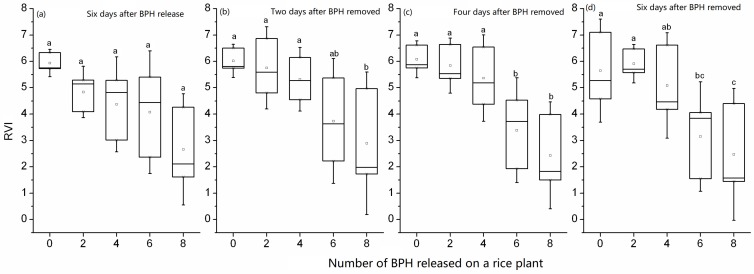
RVI_746/670_ of rice leaves exposed to 0–8 second-instar BPH nymphs for six days (**a**) and 2–6 days after BPH exposure (**b**, **c** and **d**). In the box plot, the box represents the 25th and 75th percentiles; whiskers represent the minimum and maximum values; the line in box represents the median percentile; the open circle in a box represents the mean. The different letters above the box plots show significant differences in the RVI_746/670_ means among different numbers of BPH at *p* = 0.05 level.

When BPH nymphs damaged rice plants for 6 days, the RVI_746/670_ of rice plants decreased (*F*_4, 10_ = 3.35, *p* = 0.055, Figure 5a). Two days (*F*_4, 10_ = 3.64, *p* = 0.044, Figure 5b), four days (*F*_4, 10_ = 7.15, *p* = 0.005, Figure 5c), and 6 days (*F*_4, 10_ = 4.28, *p* = 0.028, Figure 5d) after exposure to six or eight BPH nymphs, the RVI_746/670_ values of rice plants were significantly lower than that of the control. The post-effect of BPH damage on rice had been detected by the RIV_746/670_. Due to the obvious overlapping ranges, the spectral index RVI_746/670_ could not distinguish the damage of two and 4 BPH nymphs on a rice plant in six days. The RVI_746/670_ had a two or four-day delay for detecting damages of six and eight BPH nymphs in six days on a rice plant (Figure 5).

**Figure 6:**
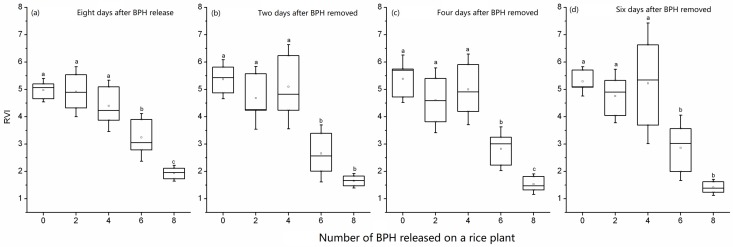
RVI_746/670_ of rice leaves exposed to 0–8 second-instar BPH nymphs for eight days (**a**) and 2–6 days after BPH exposure (**b**, **c** and **d**). In the box plot, the box represents the 25th and 75th percentiles; whiskers represent the minimum and maximum values; the line in box represents the median percentile; the open circle in a box represents the mean. The different letters above the box plots show significant differences in the RVI_746/670_ means among different numbers of BPH at *p* = 0.05 level.

When a rice plant was exposed to 6 and 8 BPH nymphs for 8 days, its spectral index RVI_746/670_ decreased significantly (*F*_4, 10_ = 20.69, *p* < 0.01, Figure 6a). Two days (*F*_4, 10_ = 17.05, *p* < 0.01, Figure 6b), 4 days (*F*_4, 10_ = 19.45, *p* < 0.01, Figure 6c) and 6 days (*F*_4, 10_ = 12.91, *p* < 0.01, Figure 6d) after BPH exposure, the values were still significantly lower than that of the control. However, the damages of two and four BPH nymphs per plant in eight days could not be identified from the healthy rice using the RVI_746/670_ (Figure 6).

Overall, the BPH density and exposure day significantly affected the ratio vegetation index RVI_760/674_ from rice plants (Table 1). These two factors also had a significant interaction on RVI_760/674_. More BPH and longer exposure day would generate the lowest RVI_760/674_ of rice. The recover day had no significant effects on RVI_760/674_, and it had no interactions with BPH density and exposure day (Table 1), suggesting that the BPH damage of rice could not recover.

### 3.2. Relationships between Spectral Index and the Number of BPH or Exposure Day

**Figure 7:**
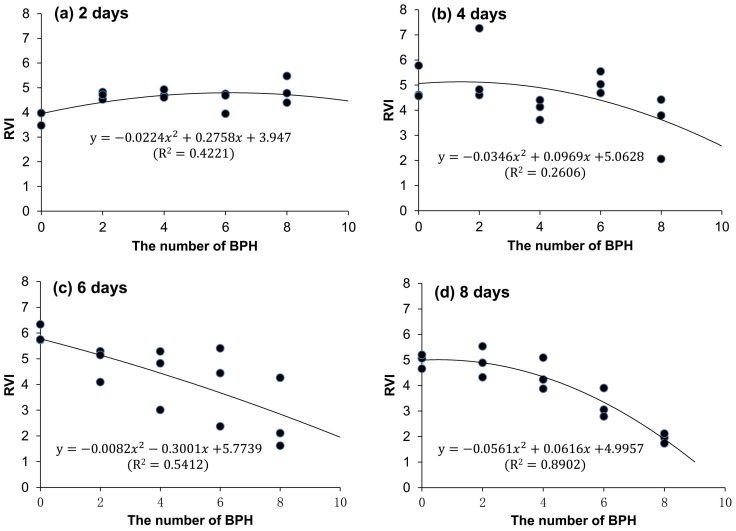
Relationships between RVI_746/670_ of a rice plant and the number of BPH nymphs damaged rice for 2 (**a**), 4 (**b**), 6 (**c**), and 8 (**d**) days.

The spectral index RVI_746/670_ from a rice plant had a quadratic curve relation with the number of BPH nymphs. This curve relation was significant for the two days (*F*_2, 12_ = 4.382, *p* = 0.037, Figure 7a), six days (*F*_2, 12_ = 7.077, *p* < 0.01, Figure 7c), and eight days (*F*_2, 12_ = 48.631, *p* < 0.01, Figure 7d) of exposure, except four days (*F*_2, 12_ = 2.114, *p* = 0.163, Figure 7b). The RVI_746/670_ (Y) from the rice plants exposed to BPH for eight days could be well evaluated based on the number of BPHs (N): Y = −0.0561N^2^ + 0.0616N + 4.9957 (R^2^ = 0.8902, *p* < 0.01). The RVI_746/670_ is sensitive to the population size of BPHs on rice plants.

**Figure 8:**
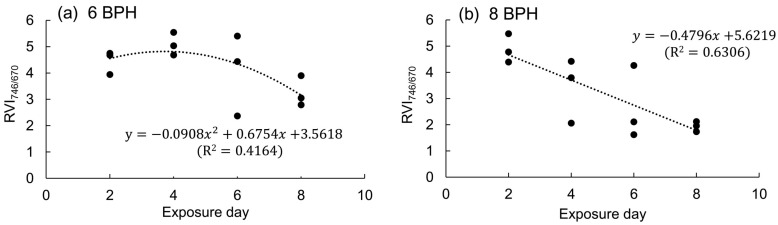
RVI_746/670_ of a rice plant exposed to 6 (**a**) and 8 BPH nymphs (**b**) for 2–8 days.

The RVI_746/670_ from a rice plant decreased as the exposure day to 6 and 8 BPH nymphs extended from two to eight days (Figure 8). The relations between RVI_746/670_ from a rice plant and exposure day could be simulated by a quadratic curve for the exposure to 6 nymphs (*F*_2, 9_ = 3.21, *p* = 0.089; Figure 8a) and by a linear model for the exposure to eight nymphs (*F*_1, 10_ = 17.07, *p* < 0.01; Figure 8b). The spectral index RVI_746/670_ of rice plants is sensitive to exposure day to BPH.

### 3.3. Model of RVI746/670 Based on the Number of BPH and Exposure Days

**Figure 9:**
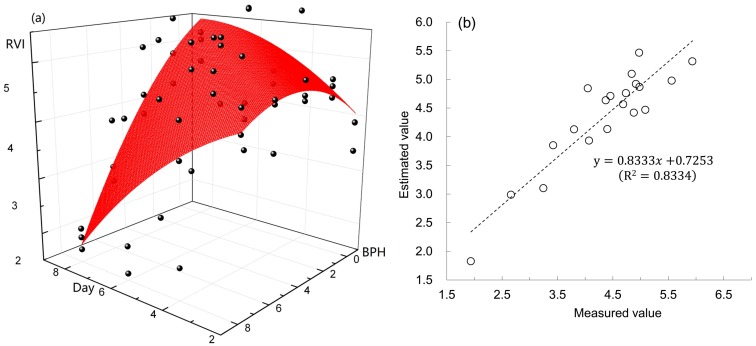
The quadratic surface model for simulating the relationships between RVI_746/670_, the number of BPH and exposure day (**a**), and the linear relationship between estimated RVI_746/670_ values based on the model and measured values from rice plants (**b**).

The RVI_746/670_ (Y) of a rice plant was strongly dependent on the number of BPH (N) and exposure days (T). It could be simulated by a quadratic surface model: Y = 3.09427 + 0.59111T + 0.44296N − 0.03683T^2^ − 0.03035N^2^ − 0.08188NT (R^2^ = 0.5228, *p* < 0.01). The model showed that a short-term damage by a low density of BPH would lead to an increase of the RVI_746/670_ from rice plants, but a high density would directly decrease the RVI_746/670_ (Figure 9a). The estimated values of RVI_746/670_ based on the quadratic surface model had a significant linear relation with the observed values (R^2^ = 0.8334, *p* < 0.01; Figure 9b). The spectral index RVI_746/670_ is sensitive to the damage of BPH on rice. Therefore, the RVI_746/670_ has the potential application to monitor damage scales of rice caused by BPH.

## 4. Discussion

The ratio vegetation index RVI has the potential to indicate the stress level of crops [7,12,19], due to its high correlation with the leaf area, dry biomass and chlorophyll content [20]. The damage degree of a crop by pests is determined by the pest population size and exposure time. The results in this study showed that the ratio spectral index RVI_746/670_ from rice plants significantly decreased with the increase of population size of BPH nymphs and the extension of exposure day. Therefore, this spectral index RVI_746/670_ has the potential to reflect the magnitude of pest stress on rice plants. Previous studies showed that the spectral reflectance from crops in red (640–740 nm) and near-infrared bands (740–1300 nm) was significantly related with the damage degree of sucking insect pests [5,13,18,22]. In this study, we confirmed that the reflectance at 670 nm and 746 nm from rice plants could indicate the BPH damage and established the spectral index RVI_746/670_. The wavebands and index may be useful to set up an air- or space-born sensor to monitor pests. On the other hand, the specific bands determined in this study will help us to interpret the remote sensing image and dig information for monitoring pests in agriculture. Therefore, the pest monitor in agriculture based on remote sensing is likely to become true in future when we find the key reflectance bands and the threshold of a spectral index like RVI_746/670_ between the damaged and healthy crops. It is noteworthy that the sensitive spectral index to BPH found in this study was derived from a rice plant, so the minimum spatial resolution of the index needs to be determined before application in airborne or spaceborne sensors, as well as the effects of size and sparsity of the affected plantations.

The sensitivity of a spectral index to damage severity of crops is a key factor to evaluate the use perspective of this index in field. The more sensitivity of an index to a slight change of damage on crops, the higher the ability to identify pest levels this index has. Many studies have indicated that the spectral reflectance at sensitive bands and their combinational indices from crops were significantly related to pest damages [5,8,23,24,25], but studies on the sensitivity and threshold of spectral index to distinguish the damage level of crops are little. Therefore, remote sensing end-products related to hyperspectral reflectance are still used less in the pest monitoring in field. A previous study suggested that the spectral index might distinguish the infested and healthy wheat when aphids had been infested for more than a threshold day, and the threshold was 25 days after aphid infestation for the winter experiment and 13 days for the spring experiment [5]. A back-propagation neural network based on full spectra from 380 nm to 1 030 nm could classify the rice plants infested with striped stem-borers *Chilo suppressalis* Walker for four or more days, but the rice damaged for two days were confused with the healthy ones [26]. The rice planthoppers are small and usually hide in the lower part of rice plants. They are easy to be overlooked. When the damage symptom caused by planthoppers appears in rice, a huge loss of rice yield has become unavoidable [27,28,29]. Therefore, the early monitoring of planthopper populations is important for the timely control. In this study, we found that the spectral index RVI_746/670_ could well distinguish the rice plants damaged by six and eight 2rd-instar BPH nymphs for eight days before appearance of visibly damage symptoms. The time threshold of RVI_746/670_ to monitor 2rd-instar nymphs would be 8 days after BPH infestation onto the tillering stage of rice. The damage of young nymphs of BPH on rice is lower than that of the old ones [28], so the time threshold of RVI_746/670_ will be earlier than eight days if the BPH population in field includes both the young and old nymphs. Moreover, the eight-day-exposure to two and four young BPH nymphs could not result in significant changes of the RVI_746/670_ from rice plants. Thus, the density threshold of BPH which RVI_746/670_ could identify would be more than four young nymphs per plant. The RVI_746/670_ could identify rice plants exposed to six and eight young BPH nymphs for eight days, which suggests that this spectral index is sensitive to BPH damage on rice and it will be applied in the population monitor.

There was a quadratic curve relation between the ratio vegetation index RVI_746/670_ from rice plants and the number of BPH nymphs on rice. Moreover, the relationships among RVI_746/670_, number of BPH and exposure day could be simulated by a quadratic surface model. These results showed that rice plants fed by a small amount of BPH nymphs for a short time (two–four days) would increase the ratio vegetation index (Figure 3a), but if the number of BPH was large, the index decreased fast. The increase of RVI_746/670_ might result from the physiologic compensation of rice plants which had been found in crops [29]. The compensation effects of crops induced by the feeding of a small number of pests can improve the performances of crops and increase yields [30,31]. Therefore, the increase of RVI_746/670_ in rice plants exposed to two and four BPHs for two days is not a consequence of inaccuracies in the measurements, but indicates a normal physiological trait of rice plants. This result further proves that the RVI_746/670_ can indicate a slight change of rice, suggesting its sensitivity to BPH damage. 

While the RVI_746/670_ did not decreased at once when rice plants were exposed to BPHs for four and six days, it significantly reduced two to six days later. The result showed that the post-effect of BPH feeding on rice plants could be identified by the spectral index RVI_746/670._ The spectral index RVI_746/670_ is sensitive to reflect the changes of rice plants during and after BPH infestation. Moreover, we established the quadratic surface model to evaluate the RVI_746/670_ of rice plants damaged by BPH for different days. Based on the model, we can assess the population size of BPH via measuring the RVI_746/670_ and occurrence time of BPH in rice field. To our knowledge, this is the first time a quadratic surface model has been set up to monitor pests based on a spectral index.

The experiments in this study were carried out under a controlled condition in the laboratory. Three replications including 60 potted rice were sufficient for finding the relationships between BPH, exposure day and RVI_746/670_. Of course, if more replications were performed, we could test the accuracy of models to evaluate the number of BPH on rice. This study focused on determining the sensitivity of a spectral index to BPH damage. The results will be used in rice field to monitor BPH. Therefore, more experiments and replications must be carried out in rice field in future. In the field, the age structures of BPH populations are various. The number of BPH does not present wholly their damage like this study. Therefore, a simplified damage level based on rice symptoms has been used [22], and the relationship between the damage level and RVI needs to establish in the further experiments.

## 5. Conclusions

BPH damage results in significant changes of the ratio vegetation index RVI_746/670_ of rice plants. RVI_746/670_ has a 2–4 days delay to detect a small number of BPHs on rice plants, but it can indicate the slight post-effect of BPH damage and the compensation effect of rice. The spectral index can exhibit the real performance of rice plants damaged by BPHs. RVI_746/670_ has a quadratic curve relation with the number of BPH nymphs on rice, and it has a quadratic surface relation with the number of BPH nymphs and exposure days. The ratio vegetation index RVI_746/670_ is sensitive to BPH damage and has the potential to monitor BPH.

## Figures and Tables

**Table 1:** Effects of BPH density, exposure day, and recover day on the RVI_760/674_ from a rice plant based on a repeated measure ANOVA.

Source	Degree of Freedom	Mean Square	*F*	*p*
BPH density	4	44.88566	41.37604	<0.001
Exposure day	3	10.27968	9.475905	<0.001
Recover day	3	1.048373	1.239454	0.298
BPH density*exposure day	12	6.314903	5.82114	<0.001
BPH density*recover day	12	0.774209	0.915321	0.534
Expousre day*recover day	9	0.929848	1.099327	0.368
BPH density*exposure day*recover day	36	0.256511	0.303264	0.999
